# Buffalo City Hospital

**Published:** 1848-01

**Authors:** 


*Buffalo City Hospital.*—During the last session of the Legislature a charter was obtained for a Hospital to be located in this city, under the above title. The Hospital has been organized by the adoption of a code of by-laws and regulations, and by the appointment of officers. The Board of Directors have taken the matter actively in hand, and are zealously prosecuting the work. Thus far, we are gratified to be able to say, that their exertions have met the prompt and unqualified approbation and co-operation of our citizens. The following memorial to the state legislature is now in circulation for the purpose of obtaining signatures:

*To the Honorable Legislature of the State of New-York* :—

Your memorialists, citizens of Buffalo, in the county of Erie, State of New-York, respectfully represent, that like other commercial cities, and especially where the population is unsettled and increasing, our city has always a large proportion of poor and sick citizens who are thrown upon the public charity, and who become a public tax.

But in addition to this, situated as we are at the junction of four or five of the most thronged highways in the State, our city is now, and has been for some years past the grand depot for a vast number of foreign immigrants, many of whom, being detained here by poverty, sickness and various other casualities, fall upon us for medical aid and support.

The exact number who pass through Buffalo, on their way to the West, we have no means of ascertaining, but your Honorable body will at once see that the number must be immense, since not only those who enter the ports of New-York and Boston, but also those who come by the way of the St. Lawrence and the Canadas concentrate upon us. And since the passage of late laws by the Congress of the United States, regulating and restricting the number of immigrants in each vessel entering our ports to a certain per centage on the tonnage, the proportion who enter by the Canadas has greatly increased. Thus New-York and Boston have been relieved of the crowd which would otherwise have pressed upon them, while the tide hitherward has not only not diminished, but has come upon us through the Canadas bringing ten fold more destitution and disease. During the past summer our County Alms House has been insufficient even to shelter comfortably the infirm paupers, and two new buildings have been devoted to the sick. More than six hundred cases of ship fever were brought among us, beside a large amount of other sickness.

We anticipate for the coming year certainly, no abatement of this burden.

We farther represent that while the city of New-York has under a liberal and enlightened policy, received for its various charities, not less than one million of dollars, directly or indirectly from the State Treasury, and is, still receiving constantly large appropriations, the western portion of the State, has never received a dollar for a similar purpose.

We believe that your Honorable body in pursuance of the same humane policy which has prompted you to sustain the New-York Hospital, and many other excellent charities in that city, from the public treasury—to some of which you have granted almost princely endowments, will also influence you to endow and sustain one similar institution in the west.

And although we have been tardy in urging our necessities, and in presenting our claims, we respectfully pray, that the fact may not be construed into an admission on our part that the necessities and claims have not long existed, but that, on the contrary, our forbearance may now ensure from your Honorable body a more prompt and liberal consideration.

We, there, your memorialists humbly pray that you will grant to the Hospital of the city of Buffalo, chartered by act of Legislature, November 1847, the sum of $40,000 for the purchase of a site and erection of suitable buildings, and the farther sum of $2000 annually during five years, for the purpose of endowing and sustaining the same.

The main grounds upon which the citizens base their claims are thus briefly set forth, but the Memorial has not attempted to go at length into the several arguments. The following statement, however, made officially by Mr. L. Brace, one of the superintendents of the poor for this county, is exactly in point:

"In the year commencing 1843, Oct. 1st and ending Oct. 1st, 1844, the Superintendents of the Poor of Erie county reported to the Secretary of State, that there had been received into the poor house 675 persons. The expenses of supporting them was $7,257 72; and that they had expended in temporary relief $10 167 25—(the number of persons temporarily relieved was not given)—making the whole expenses $17,424 97.

From Oct. 1st, 1844, to Oct. 1st 1845, the whole number of paupers relieved or supported was 2647; number temporarily relieved was 1901; expenses at the county poor house were $7,074 85, and for temporary relief $8,192 28—making the sum of $15,267 13.

From 1st of Oct., 1845, to 1st Oct., 1846, the whole number relieved or supported was 2,841. Expenses at the poor house were 5,938 99; for temporary relief, $9,154 77; making $15,093 76.

From Oct. 1st, 1846, to Oct. 1st, 1847, the whole number of paupers relieved or supportedwas 4,301. The number of all such persons who were temporaly relieved was 3,292. The aggregate expense of relieving and supporting all such persons was $17,146 83; of this aggregate sum, the amount expended for tempory relief was $8,367 24; poor house expenses, $8,779 59. The whole number received into the poor house during the last year was 978: died in the poor house, 113. It may be added here that the City Sexton buried during the same time 218, by request of the Overseers of the Poor. It will readily be seen that while pauperism has been annually on the increase, that at no time does its increase compare with the last year, there having been in that time an addition of a little less than fifteen hundred, all of whom were in a most miserable, destitute and sickly condition. Scarcely a family that has stopped here since last July, but more or less of its members have brought with them ship fever; and I am fully satisfied that one thousand have been proper subjects for a hospital.

Of the 4201 paupers the last year, 3804 were foreigners, viz: 1781 from Ireland; 1265 from Germany; 198 from England; 113 from Scotland; 92 from Holland; 71 from France; 47 from Canada; 18 from Prussia; 16 from Wales, 6 from Halifax; 74 colored, and 529 native citizens.

Besides these, from 800 to 1000 who have passed through this city, have been more or less aided, and of whom no official record has been kept.

Is it just and equal that this city and county should be subjected to this enormous tax for the support of foreign paupers? The Legislators have evidently believed that it is not just or equal, and for the purpose of remedying the evil, imposed a tax upon all imigrants entering the port of New-York city, of one dollar per head. During the year ending Sept. 30, 1847, 145,830 landed in New-York, and if the per cap. tax was actually received, it has in this one year amounted to $145,830. This money passes into the county treasury, or into the hands of the county superintendants, who hold it in trust for the Co. of N. Y., and such other counties of the State as may be able to give the required evidence that within a certain time any of the imigrants who have paid said tax, have become a charge to the county. This is, we are informed, the substance of the law, but unfortunately for us who annually support such a large number of these immigrants, its practical working is not what the legislators designed it should be, since it is found almost impossible to identify these paupers when once they have gone out from the county of New-York; and, so great is the difficulty that during the last year not more than two or three families have been identified here as having entered and commuted at the port of New-York. New-York city has thus been left in the almost undisturbed possession, so far at least as we are concerned, of this entire sum; and it has received in addition for the maintenance of its immigrant hospitals from the Treasury of the State, the sum of ten thousand dollars.

During the session of the Legislature just closed, another similar statute has been enacted, demanding a tax of one dollar per head upon all such as enter the State of New-York north and west of Albany, along the line of the St. Lawrence, Lake Ontario, Niagara River, and Lake Erie; which tax is also to be paid to the superintendents of the poor in the county in which they shall make their entry, and if within three years thereafter any of these persons become a county charge elsewhere in the State, such other county may draw upon the county where they paid their commutation, for the amount of their expenses, until the whole amount received is paid out. But the same cifficulty still exists; they cannot be identified, and the county in which they land will be alone benefitted; by which rule the amount of benefit derived to this county must be fractional.

We are not prepared to say what remedy for this evil can be devised, but it is certain that so far as this, and most other counties of the State are concerned, the law is worthless, and it would be more equal for us if the whole amount was paid directly into the general treasury.

There can be no doubt that the amount of foreign pauperism in this city is greater in proportion to the population than in any other city in the State, and while New-York city may have an equitable claim on the State for all or nearly all of the immigrants tax and ten thousand dollars directly from the State Treasury for the maintenanc of this class of paupers, we also have an equitable claim for even a larger proportion of the public funds, since we have vastly less pecuniary ability.

We have been led to make these remarks at this time in order that the members of the profession in the western part of the state may understand the merits of our application, and by representing the facts to their several representatives, co-operate with us in giving to the west its due.

According to the report of the Treasurer, the city of New-York received, during the last fiscal year, from the general state fund, and from the United States deposite fund, for its various charitable institutions, $77,268,47; other sums were also received from other funds. And similar sums are paid annually for the same purposes. What charitable institution out of New-York has, as yet received one dollar?

